# Optimal Design of the Absolute Positioning Sensor for a High-Speed Maglev Train and Research on Its Fault Diagnosis

**DOI:** 10.3390/s120810621

**Published:** 2012-08-03

**Authors:** Dapeng Zhang, Zhiqiang Long, Song Xue, Junge Zhang

**Affiliations:** College of Mechatronics Engineering and Automation, National University of Defense Technology, Changsha, Hunan 410073, China; E-Mails: zhangdapeng12@163.com (D.Z.); songself@126.com (S.X.); junge@sina.com (J.Z.)

**Keywords:** high-speed maglev train, absolute positioning sensor, fault diagnosis, position mark plate, support vector machine

## Abstract

This paper studies an absolute positioning sensor for a high-speed maglev train and its fault diagnosis method. The absolute positioning sensor is an important sensor for the high-speed maglev train to accomplish its synchronous traction. It is used to calibrate the error of the relative positioning sensor which is used to provide the magnetic phase signal. On the basis of the analysis for the principle of the absolute positioning sensor, the paper describes the design of the sending and receiving coils and realizes the hardware and the software for the sensor. In order to enhance the reliability of the sensor, a support vector machine is used to recognize the fault characters, and the signal flow method is used to locate the faulty parts. The diagnosis information not only can be sent to an upper center control computer to evaluate the reliability of the sensors, but also can realize on-line diagnosis for debugging and the quick detection when the maglev train is off-line. The absolute positioning sensor we study has been used in the actual project.

## 1. Introduction

**Figure 1.**
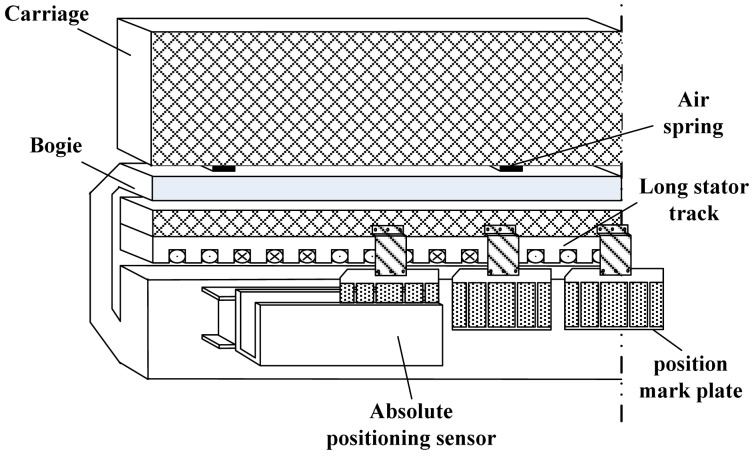
The structure of the absolute positioning sensor on the maglev train.

The high-speed maglev train moves without any contact with the tracks depending on the electromagnetic force levitation, and it is driven by a linear synchronous motor. In order to obtain the optimal drive efficiency, the accurate electrical angle is needed when the train is running. Relative positioning sensors can achieve millimeter-sized positioning resolution, but they suffer from accumulated errors [1–3]. In order to reset the relative accumulated errors, it is necessary to use absolute positioning sensors to calibrate the relative positioning sensors at intervals [4,5]. The structure of the absolute positioning sensor on the maglev train is shown in Figure 1.

In addition, the absolute positioning sensor is working under conditions of high temperature, strong electromagnetic disturbance and high-speed horizontal movement. Due to these factors, it is almost impossible for humans to perform the fault diagnosis for the sensors directly, especially while the train is running. Based on the reasons above, how to locate the faults of the sensor reliably for upper center control computer to make evaluations, and how to realize high efficiency detection for the sensor are also new problems for us too. Aimed at solving these problems, this paper proposes an absolute positioning sensor and designs a system to realize the automatic fault diagnosis for the sensor. The experimental results show that this method can reliably accomplish the automatic fault diagnosis.

## 2. Design of Absolute Positioning Sensor

### 2.1. The Basic Principle Analysis of the Absolute Positioning Sensor

**Figure 2.**
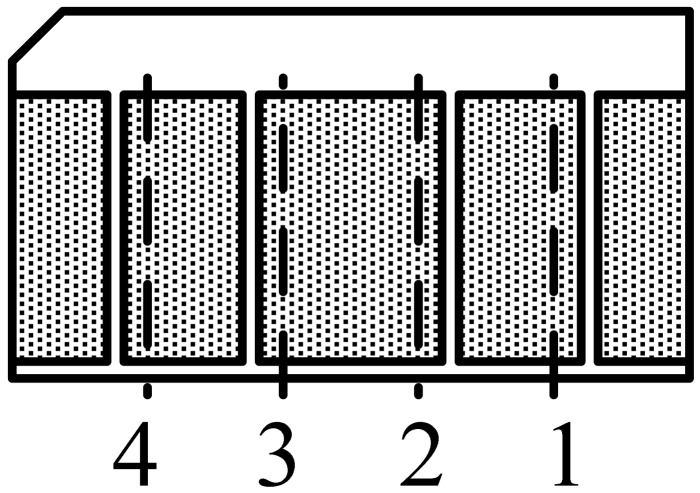
The structure of the position mark plate.

When the high-speed maglev train is moving on the track, its absolute positioning sensors read the position mark plate groups fixed on the track every 200 meters. Every position mark plate contains 4 bits of binary position information. Usually, every position mark plate group consists of three position mark plates, so the total position information is 12 bits. Thus, it can describe a distance of 800 kilometers at most. The structure of the position mark plate is shown in Figure 2.

Define that if the narrow slot is on the left side of the broken line, it presents the binary “1”, and if the narrow slot is on the right side of the broken line, it presents the binary “0”. As Figure 2 shows, the position mark plate presents binary number “1100”. In the middle of position mark plate, there is a metal foil layer. As a result of eddy current stimulated by the variational magnetic field in the metal layer, the original source magnetic field is weakened. This results in that the electromagnetic wave can only pass through the narrow slots.

**Figure 3.**
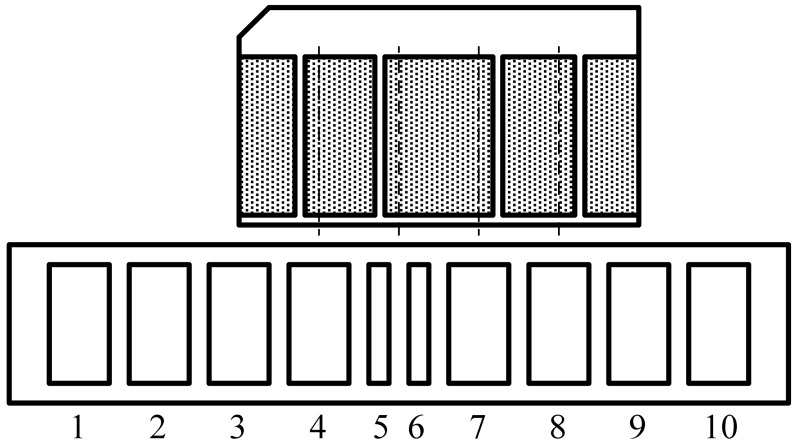
The principle of absolute positioning sensor.

The maglev train has no contact with the tracks, so the absolute positioning sensor is designed to read the position mark plate based on electromagnetic induction [6–8]. The RF coils are classified as positioning coils and code reading coils. As Figure 3 shows, 1–7, 2–8, 3–9, 4–10 are four groups of positioning coils, and 5–6 is a group of code reading coils. The reading coils are narrower than the positioning coils. The four groups of positioning coils are related to the binary codes of the No. 1 to No. 4 narrow slots (see Figure 2). For example, when the position mark plate is at the position shown in Figure 3, the board shields half of the 3–9 coil group. At this time, the voltage induced by the receiving coils just passes the zero point, and at the same time, the digital circuit starts to read 5–6 coil group's state. If the induced voltage on the No. 5 coil is larger than that on the No. 6 coil, the 5–6 coil group's differential voltage is positive. Otherwise, the 5–6 coil group's differential voltage is negative. Thus, the sensor can read out the binary information in the No. 3 narrow slot.

### 2.2. The Optimal Design and the Realization for the Absolute Positioning Sensor

**Figure 4.**
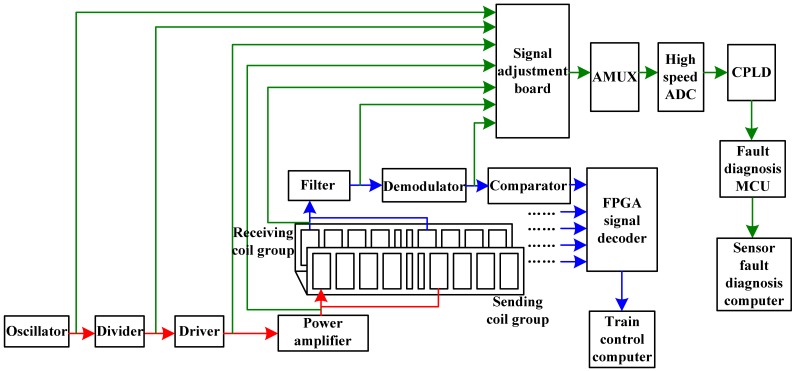
The hardware structure of absolute positioning sensor.

The absolute positioning sensor consists of five channels of circuits which almost have the same hardware structure. Every channel of the circuits includes a RF power amplification circuit and a signal receiving adjustment circuit. The hardware structure is shown in Figure 4. The figure shows only one channel among the five, and the other four are the same. Class C power amplifiers are used to drive the sending coils. According to the center frequency of the resonance, band-pass filters are designed for the receiving side. The induced voltage stimulated by the electromagnetic wave which passes through the narrow slot is picked up by the method of synchronous demodulation. The induced voltage is used to compare with a certain threshold value to get the rising edge or the falling edge which means the positioning coil is ready to locate. At last, based on the five channels' signals, the high-speed FPGA chip decodes the binary information with finite state machines [9,10]. After finishing reading the whole group of position mark plate, it sends the information to the upper computer.

#### 2.2.1. Design of the Sending and Receiving Coils

As we know, the magnetic induction intensity is mainly distributed on the receiving coils which are right facing their sending coils, but usually there still exist effects to the other coils nearby. Therefore, the interactive electromagnetic field stimulated by the five channels of sending coils may disturb the five channels of receiving coils. So, in the design, five different frequencies are used between 1 to 3 MHz. Every group of sending coils is driven by a signal source with different frequency.

**Figure 5.**
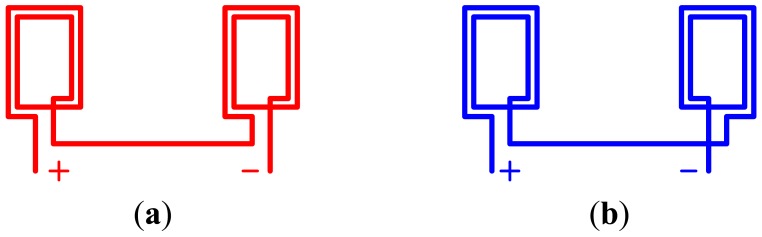
The structure of the sending coils and the receiving coils in a group. (**a**) The sending coils; (**b**) The receiving coils.

The structures of the receiving coils and sending coils are shown in Figure 5. In order to make the coils in the same group stimulate the same directional electromagnetic field, the sending coils are wound in the same direction and connected in series. While the high-speed maglev train is running, there is very strong electromagnetic disturbance generated by the synchronous driving linear motor around the sensor. In order to reduce the disturbance, the receiving coils in the same group are wound in the different direction and connected in series. This method enhances the valid signals and weakens the disturbance signals.

#### 2.2.2. The Design of RF Power Amplifiers

**Figure 6.**
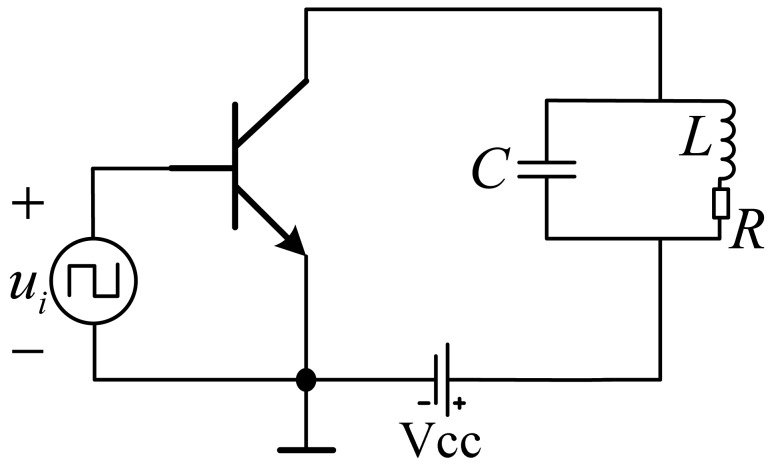
The topology structure of class C amplifier.

The RF power amplifiers can be classified as class A, class AB, class B or class C on the basis of conduct angles. Because of the high wastage of class A power amplifiers, they are often used in low power situations. Class AB, class B and class C are all suitable for use in high power situations. However, among them, the efficiency of class C is the highest one and it is proper apply to the circuit which is used to detect the variation of the carrier wave's amplitude. The absolute positioning sensor can implement its function just by detecting the amplitude of the induced voltage of the receiving coils. Therefore, the class C amplifier is proper to use in the sensor. The topology structure of a class C amplifier is shown in Figure 6.

The output power of the class C power amplifier is:

(1) $$P_{o}=\frac{1}{2}I_{Lm}^{2}R_{L}=\frac{I_{cm}^{2}R_{L}}{8\pi^{2}}{\left(2\theta -\sin2\theta \right)}^{2}=\frac{V_{cm}I_{cm}}{4\pi }(2\theta -\sin2\theta )$$

The power supplied by the source is:

(2) $$P_{s}=V_{cc}I_{c0}=\frac{V_{cc}I_{cm}}{\pi }(\sin\theta -\theta \cos\theta )$$

Then the efficiency of the class C power amplifier is:

(3) $$\eta =\frac{P_{o}}{P_{s}}=\frac{\frac{1}{2}V_{cm}I_{Lm}}{V_{cc}I_{c0}}=\frac{V_{cm}(2\theta -\sin2\theta )}{4V_{cc}(\sin\theta -\theta \cos\theta )}$$

Depending on the measurement in the fact circuit, set *V_cc_* = 24 *V, θ* = 1.3 *rad*. The designed efficiency is *η* = 92%.

#### 2.2.3. Design of the Filters

**Figure 7.**
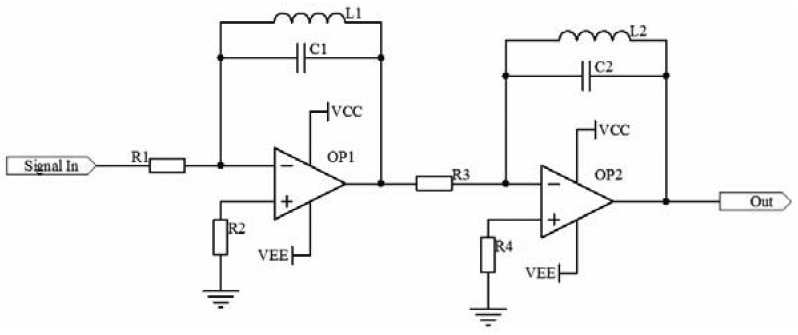
The schematic diagram the band-pass filter.

When the receiving coils are wound as it is shown in Figure 5(b), they can offset the external common mode disturbance to some extent, but they are useless for the electromagnetic disturbance stimulated by the nearby sending coils, so filters are needed in the circuit. Because of the center frequency of the carrier is somewhat high, if filters based on the topology structure of VCVS or Sallen-Key are designed, the gain bandwidth of the operational amplifiers will be required to be very high when a high Q value filter is needed. In the actual circuit, this is really hard to implement. Hence, this paper designed a band-pass filter with inductances and capacitors to process the received signal. The schematic diagram is shown in Figure 7.

The transfer function of the filter is:

(4) $$W(s)=\frac{\frac{L_{1}}{R_{1}}s}{L_{1}C_{1}s^{2}+1}\cdot \frac{\frac{L_{2}}{R_{3}}s}{L_{2}C_{2}s^{2}+1}=\frac{\frac{L_{1}L_{2}}{R_{1}R_{3}}s^{2}}{L_{1}C_{1}L_{2}C_{2}s^{4}+(L_{1}C_{1}+L_{2}C_{2})s^{2}+1}$$

The gain of two stages four orders filter is related to the characteristic impedance of the parallel connection of LC denoted by *Z_F_*. The gain is:

(5) $$A_{u}=\frac{Z_{F1}}{R_{1}}\cdot \frac{Z_{F2}}{R_{3}}$$

From Equation (5), the gain of the filter can be adjusted conveniently by selecting the values of *R_1_* and *R_3_* properly.

**Figure 8.**
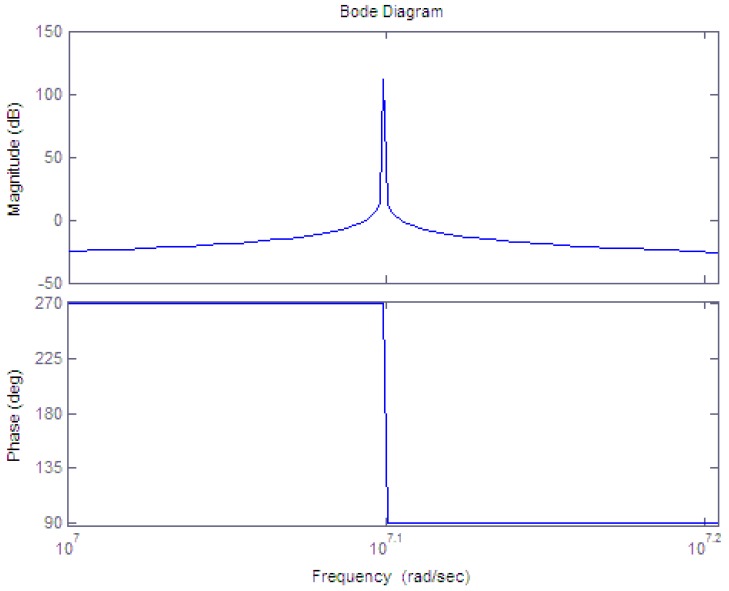
The bode diagram of one stage of the filter.

The performance of the band-pass filter is determined by the Q value of LC components. The higher the Q value is, the better the performance of frequency selecting. If set the center frequency of the band-pass filter to *f_o_* = *2 MHz, L_1_* = *20 μH, C_1_* = *317 pF, R_1_* = *10 kΩ*. The Bode diagram of one stage of the filter with Matlab is achieved. It is shown in Figure 8.

**Figure 9.**
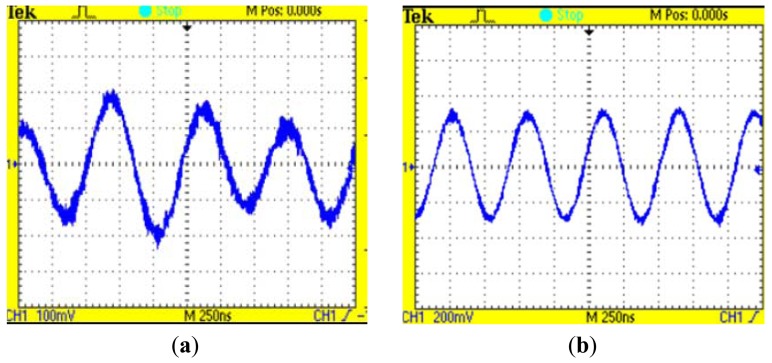
(**a**) The signal before filtering; (**b**) The signal after filtering. The axes in *x* direction presents the time and the axes in *y* direction presents the voltage of the signal.

The comparison of the signals before and after filtering is shown in Figure 9.

#### 2.2.4. The Design of the Demodulation Circuit

**Figure 10.**
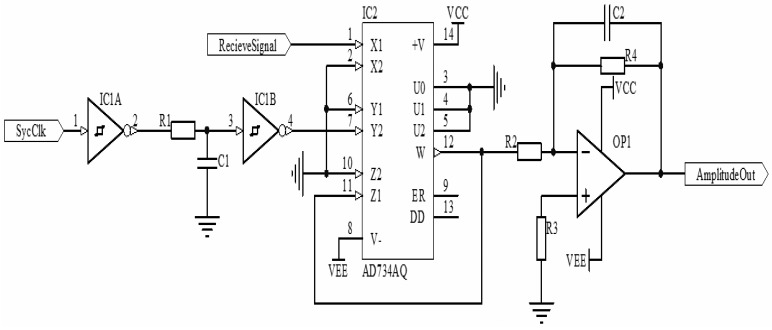
The schematic of synchronous demodulation.

As the analysis above, the amplitude of the induced voltage of the receiving coils should be used to locate the position of the position mark plate, so the the signal must be demodulated after filtering. The demodulation methods that are usually used are square demodulation, envelope demodulation and synchronous demodulation. Considering the special differential structure of the receiving coils, when the positioning coils accomplish locating, the phase of the induced voltage will reverse. If multiplying the received signal by the synchro clock, the variation of the induced voltage can be detected. In addition, the sending circuit and the receiving circuit are both on the same board, so the synchronous demodulation method based on a 4-quadrant multiplier is selected by this paper. The schematic is shown in Figure 10.

**Figure 11.**
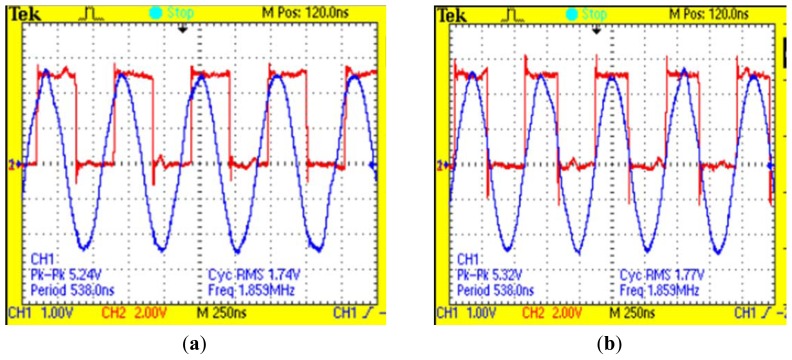
The comparation of the sychro square clock signal before the phase adjusts and after the phase adjusts. (**a**) The input signals before the phase adjusts; (**b**) The input signals after the phase adjusts.

The PN junction of the power transistor contains parasitic capacitance and the receiving signal will pass through a band-pass filter. Therefore, there exists some delay between the clock source signal and the signal after filtering. The phase difference will decrease the precision of the amplitude detection. In order to offset the phase difference, a RC delay circuit with Schmitt-trigger inverters is designed. If the RC value is adjusted properly, the synchro clock signal and the signal after filtering will be adjusted to zero phase error state. The result of phase adjustment is shown in Figure 11.

As Figure 11 presents, the red waveform is the sychro square clock signal and the blue waveform is the receiving signal after filtering. They are both the multiplier's input signals. Before adjustment, there exists severe phase difference between the receiving signal and the synchro signal, but after the adjustment, the phase difference is offset.

The label ‘RecieveSignal’ in Figure 10 is the receiving signal after filtering, and the label ‘SycClk’ is the synchro clock signal. The operational function of 4-quadrant multiplier is:

(6) $$W=-X_{1}Y_{2}/10$$

where *X_1_* and *Y_2_* are the input signals which are mentioned above. We denote the signal after filtering by *V_r_*, and its frequency by *ω_0_*, amplitude by *A_r_*; denote synchro square clock signal by *V_syc_*, and its frequency by *ω_0_*, amplitude by *A_syc_*. Suppose that the disturbance signal generated by the nearby sending coils is *V_d_* = *A_d_*sin*(ω_d_t* + *θ)*, and generally speaking, *ω_d_* ≠ *n*·*ω_0_*. The receiving signal in total is denoted by *V_tr_*, then:

(7) $$V_{tr}=V_{r}+V_{d}=A_{r}\sin(\omega_{0}t)+A_{d}\sin(\omega_{d}t+\theta )$$

The square clock signal can be decomposed as:

(8) $$V_{\text{syc}}=\frac{A_{\text{syc}}}{2}+\frac{2A_{\text{syc}}}{\pi }\sum_{n=1}^{\infty }\left(\frac{1}{2n-1})\sin[(2n-1)\omega_{0}t\right]$$

So, the output of the multiplier can be inferred as follows:

(9) $$\begin{array}{l} W=(V_{tr}\cdot V_{\text{syc}})/10\\ =\frac{1}{10}\{\frac{A_{\text{syc}}}{2}+\frac{2A_{\text{syc}}}{\pi }\sum_{n=1}^{\infty }\left(\frac{1}{2n-1})\sin[(2n-1)\omega_{0}t\right]\}\cdot [A_{r}\sin(\omega_{0}t)+A_{d}\sin(\omega_{d}t+\theta )]\\ =\frac{A_{\text{syc}}}{20}[A_{r}\sin(\omega_{0}t)+A_{d}\sin(\omega_{d}t+\theta )]+\sum_{n=1}^{\infty }M_{n}[\cos2n\omega_{0}t-\cos(2n-2)\omega_{0}t]+\sum_{n=1}^{\infty }N_{n}\{\cos[(2n-1)\omega_{0}t+\omega_{d}t+\theta ]-\cos[(2n-1)\omega_{0}t-\omega_{d}t-\theta ]\}\\ =\frac{A_{\text{syc}}A_{r}}{10}+W_{ac} \end{array}$$

In Equation (9), $M_{n}=\frac{A_{\text{syc}}A_{r}}{10(2n-1)\pi }$, $N_{n}=\frac{A_{\text{syc}}A_{d}}{10(2n-1)\pi }$, *n* = 1,2,3,…. From the equation above, the output of the multiplier includes the DC part and the AC part. Using the active low-pass filter, the high frequency AC components *ω_0_* and *ω_d_* will be filtered and the DC part $\frac{A_{\text{syc}}A_{r}}{10}$ remains. Because the amplitude of synchro square clock signal is invariant, the value of the DC part is only related to the amplitude of receiving signal. Therefore, the synchronous demodulation enhances the capacity of disturbance rejection for the sensor.

## 3. The Fault Diagnosis of Absolute Positioning Sensor

### 3.1. Fault Locating in Signal Flow Method

The physical world is causal. Therefore, when some signal flow into a component of the sensor, the pattern character of its output always be certain in some range. According to this opinion, in order to find a fault, the pattern of a component's output can be compared with the pattern in the knowledge library which is built based on the *a priori* knowledge. We check the key node of the circuit along the direction of the signal flow in this way one component and another. If the character of the pattern coincides with the knowledge library, it is considered that the components before the key node are in good condition. Otherwise, it is considered that the component before the key node is broken-down. In this way, the position of the fault will be found if the fault exists indeed.

**Figure 12.**
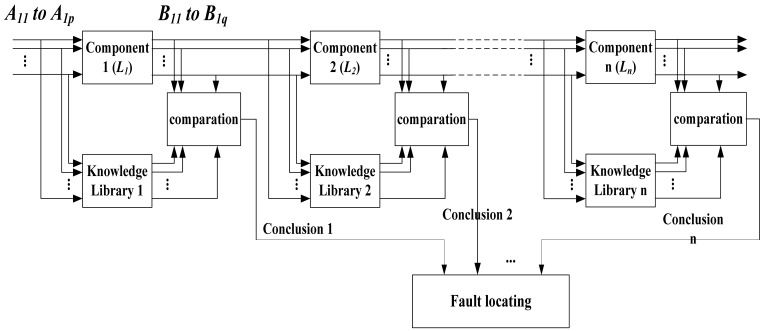
The signal flow of fault location.

The pattern of the signal that flows in and out a component can be expressed as follows:

$$\begin{array}{llll} \text{If} & L_{1}(A_{1},A_{2},\ldots ,A_{p} & \text{Then} & B_{11},B_{12},\ldots ,B_{1q};\\ \text{If} & L_{2}(A_{1},A_{2},\ldots ,A_{p} & \text{Then} & B_{21},B_{22},\ldots ,B_{2q};\\ & \;\vdots & & \\ & \;\vdots & & \\ \text{If} & L_{n}(A_{1},A_{2},\ldots ,A_{p} & \text{Then} & B_{n1},B_{n2},\ldots ,B_{nq}. \end{array}$$

where, *L_1_* to *L_n_* present the *n* groups of mapping relations between the signals that flow into the component and those flow out of the component. *A_1_* to *A_p_* present the characters of *p* input signals. *B_1_* to *B_q_* present the characters of *q* output signals. The block diagram for the signal flow of fault location is shown in Figure 12.

However, in the actual circuit, the pattern characters cannot coincide absolutely with the pattern character which is built in the knowledge library. Depending on the knowledge library absolutely may rise the false-alarm rate. Even though a threshold value can be selected to decide the range of error, yet how to select the threshold value properly is not easy. How to make the fault diagnosis reliably is a problem in the actual design. In the next section, a support vector machine method will be introduced. In this method, a component's fault state can be distinguished reliably from its normal state.

### 3.2. Support Vector Machines

Support vector machine (SVM) is a method for classification based on small specimens [11,12]. It is proposed by Vapnik and his research group in 1995. The method has been adopted rapidly in the pattern identification, fault diagnosis and statistic learning domains. It is a practical method to solve the two classes' optimal discriminant function based on the optimization idea of quadratic programming [13–15]. The method not only can solve the linear divisible problems but also can solve the linear indivisible problems on the high dimensional space when the kernel function is considered. The support vector machine is a kind of widely-used and promising method to solve the pattern classification problem [16]. The original problem of SVM can be expressed as:

(10) $$\min\Phi (w,b)=\frac{1}{2}<w,w>$$

(11) $$y_{i}(<w,x_{i}>+b)\geq 1,i=1,2,\cdots ,l$$

With Lagrange multiplier method, the corresponding dual problem is achieved:

(12) $$\max W(\alpha )=\sum_{i=1}^{l}\alpha_{i}-\frac{1}{2}\sum_{i=1}^{l}\sum_{j=1}^{l}\alpha_{i}\alpha_{j}y_{i}y_{j}<x_{i},x_{j}>$$

(13) $$\sum_{i=1}^{l}\alpha_{i}y_{i}=0,\alpha_{i}\geq 0,i=1,2,\ldots ,l$$

In Equations (10) and (11), *α_i_, i* = 1,2,…*l* are variables to be optimized, *x_i_, i* = 1,2,…*l* are the specimens to be trained, *y_i_, i* = 1,2,…*l* present the class the specimens drop in.

The linear discriminant function is:

(14) $$f(x)=\mathrm{sgn}(\sum_{SV}\alpha_{i}^{*}y_{i}<x,x_{i}>+b^{*})$$

In order to reduce the impact the linear indivisible specimens make on the result of the discrimination, add the punishment factor *C*. The problem is transformed to:

(15) $$\max W(\alpha )=\sum_{i=1}^{l}\alpha_{i}-\frac{1}{2}\sum_{i=1}^{l}\sum_{j=1}^{l}\alpha_{i}\alpha_{j}y_{i}y_{j}<x_{i},x_{j}>$$

(16) $$\sum_{i=1}^{l}\alpha_{i}y_{i}=0,0\leq \alpha_{i}\leq C,i=1,2,\ldots l$$

If the specimens are not linearly divisible in current space, according to the theory of SVM, they can be mapped from the current space to a higher dimension space. In the new space, solve the discriminant function. For example, build the mapping *x* → Φ(*x*), and the SVM problem is:

(17) $$\max W(\alpha )=\sum_{i=1}^{l}\alpha_{i}-\frac{1}{2}\sum_{i=1}^{l}\sum_{j=1}^{l}\alpha_{i}\alpha_{j}y_{i}y_{j}<\Phi (x_{i}),\Phi (x_{j})>$$

(18) $$\sum_{i=1}^{l}\alpha_{i}y_{i}=0,0\leq \alpha_{i}\leq C,i=1,2,\ldots l$$

At this time, the discriminant function is:

(19) $$f(x)=\mathrm{sgn}(\sum_{SV}\alpha_{i}^{*}y_{i}<\Phi (x),\Phi (x_{i})>+b^{*})$$

Define the symbol *k(x_i_, x_j_)* = <Φ(*x_i_*), Φ(*x_j_*)>, and *k*(*x_i_, x_j_*) is the kernel function. With the kernel function, the bother of identifying the style and the parameters of Φ(*x*) is avoided. Instead, only a proper norm in Hilbert space is needed to be found. This makes the classification job become much simpler.

### 3.3. Selection of the Character

In order to locate the fault exactly, the selection of the signal's character is very important. If an analog signal is periodic, the shape of the wave (sine wave, square wave, triangle wave, sawtooth wave, *etc.*), the frequency, the amplitude, the DC bias and so on are usually considered. If an analog one is nonperiodic, the energy of the signal, the frequency spectrum, burst-out character in some time period and so on are of concern.

The character of periodic signals can be picked up directly by the sampling. Although the character of nonperiodic signals seems complex, yet, the FFT, discrete wavelet transforms and some other methods can be used to realize the pick-up of the character in the actual application.

In the absolute positioning senor, most of the analog signals are periodic. In the sending and receiving resonance circuit, it is possible to generate nonperiodic signal as a result of the resonance center drift fault. In addition, the periodic signal may become a nonperiodic signal too. For these kinds of faults, FFT should be used to achieve the frequency spectrum of the signal. According to the character of frequency spectrum, whether this part of the circuit is broken down is discriminated.

Therefore, this paper selected the shape and the frequency spectrum of the signal as the characters to diagnose the faults of the sensor.

### 3.4. The Improvement of the Faults' Identification

For the circuits with series types, if the former component is broken down, all the latter components' outputs must not coincide with the expected pattern character. Thus, if the system alarms that some component is broken down, but the latter components' outputs coincide with the expected ones, the alarm is considered to be wrong.

The absolute positioning sensor's topology of the signal flow is relatively simple. Most of the signals are SISO series type. By comparing with the latter components' pattern character of an alarmed component with their expected pattern characters, more degrees of freedom to evaluate the trustful degree of an alarm are achieved.

For a alarmed key node of the sensor's circuit, suppose that the exact ratio of a SVM classification is *p_svi_, i* = 0,1,2,…*n*, where *n* is the number of latter components. *p_sv0_* is the alarmed component's exact ratio. Then the trustful degree of the alarm can be calculated as follows:

(20) $$k_{\text{fault}}=\alpha_{0}p_{sv0}+\alpha_{1}p_{sv1}+\alpha_{2}p_{sv2}+\cdots +\alpha_{n}p_{\text{svn}},\;j=1,2,\ldots ,n$$

In Equation (20), *α*_0_ = 1. If the latter nodes' identification coincide with the expected ones, *α_j_* = −1. If the results of the latter nodes' identification don't coincide with the expectation, *α_j_* = 1. If *k_fault_* >0, the alarm is believed exact. If *k_fault_* < 0, the alarm is omitted.

### 3.5. The Realization of the Fault Diagnosis Subsystem of the Sensor

**Figure 13.**
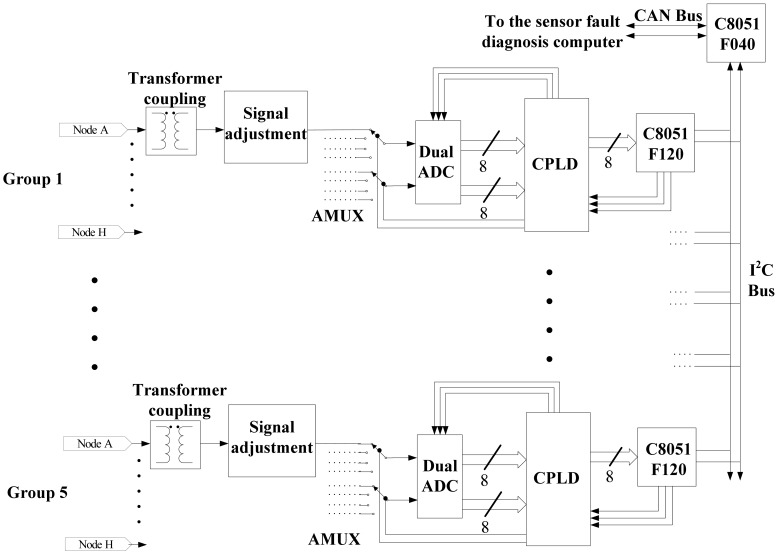
The overall hardware block diagram of the fault diagnosis subsystem for the absolute positioning sensor.

The fault diagnosis subsystem of the absolute positioning sensor also consists of five channels. They diagnose every channel of the analog circuits, respectively. The fault diagnosis subsystems in every channel of the analog circuits consist of signal adjustment circuits, analog multiplexer switches, AD converter circuits, digital signal process circuits and communication interfaces. The overall hardware block diagram is shown in Figure 13.

The input signals are coupled with the transformer. The segregated function of the transformer can decrease the influences of the original circuits' electric character. The main function of the signal adjustment circuits is enhancing the input impedance for the input signal, adjusting the amplitude of the signal and the DC bias. In order to decrease the distortion of the signal when it passes through the analog switch, the wide bandwidth analog switches, FASL200, are selected. For every channel of circuit in the fault diagnosis subsystem, there is an integrated dual AD converter, AD9288. The AD converter can take samples for two channels at one time. Because the sampling rate of the AD converter reaches 80 MHz, it is needed to use high-speed CPLD to realize the timing sequence control for the Dual ADC and the analog multiplexer switches directly and to save the sampling results to the duplicate port RAM generated in the CPLD. The digital signal process unit takes the C8051F120 as the core. Its operating speed is as high as 100 MIPS. It can accomplish the FFT quickly to the sampling data, and then on the basis of the signal flow method and the off-line trained discriminant function, C8051F120 estimates whether the nodes are in fault states.

**Figure 14.**
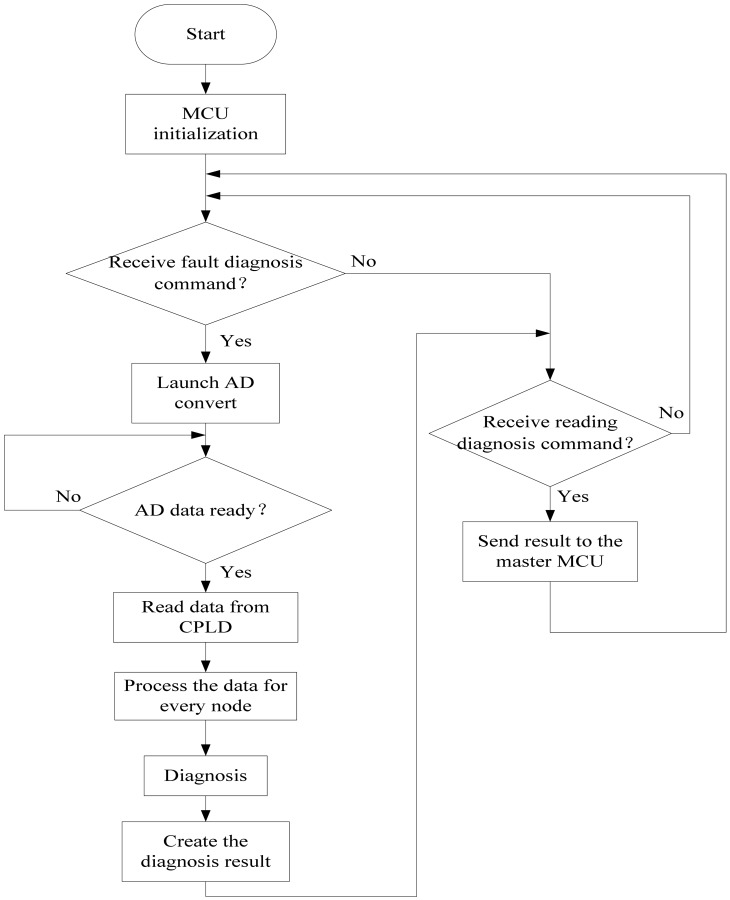
The program flow diagram of the slave MCU.

The computer control of the system is a master-slave structure. The master MCU is a C8051F040 whose task it is to communicate with the upper fault diagnosis computer and receive the commands from the upper computer. The slave MCUs are five digital signal processors that are mentioned above. The slave MCUs receive the commands from the master MCU by I^2^C bus, and then set up the AD converter to sample every key node by controlling the CPLD. Afterwards, they do the digital signal processing and the fault diagnosis. Finally, the master MCU sends out the reading result command, gathering all the results from the five channels of slave MCUs and generating a fault diagnosis datagram for the absolute positioning sensor. If required, the master MCU sends the datagram to the upper computer by the CAN bus. The program flow diagram of the slave MCU is shown in Figure 14.

## 4. The Experiment on the Ground and on the Train for the Absolute Positioning Sensor

### 4.1. The Experiment Analysis of the Absolute Positioning Sensor

#### 4.1.1. The Experiment on the Ground

**Figure 15.**
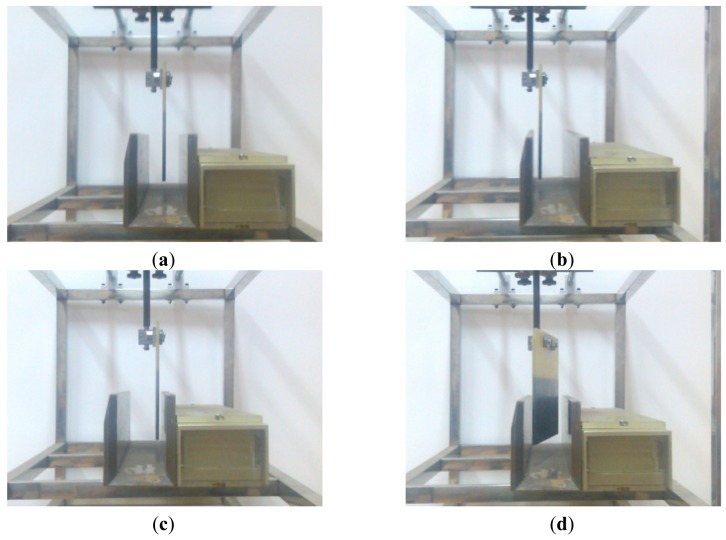
(**a**) The position mark plate is on the medium of the reading area; (**b**) The board is on the outer side; (**c**) The board is on the inner side; (**d**) The board is leaning 15 degrees.

The test situations on the test table for the sensor are shown in Figure 15. The tests include the position mark plate on the medium of the reading area, the position mark plate on the outer side of reading area, the position mark plate on the inner side of reading area and the position mark plate leaning 15 degrees. The four situations described above are all probable to occur in actual application. On the basis of the four situations, the robustness of the sensor can be tested.

The results of the experiments on the test platform are shown in Table 1.

#### 4.1.2. The Experiment on the Maglev Train

In order to test the performance under actual conditions, we tested the absolute positioning sensor on the 1.5 km long experimental maglev line in Shanghai. On the experimental line, three position mark plates are in one group. Every group is at a distance of 100 meters. The binaries of the position mark plates in the region the train can move are from 110 H to 11 BH. Three groups of data are tested, and they are shown in Table 2.

The results show that under the actual conditions, the sensor can also maintain the high reliability. The test results on the test maglev train shown that the absolute positioning sensor can calibrate the accumulated errors for the relative positioning sensor reliably so that it ensured the position signals required by the driving and running control system.

### 4.2. The Experiment of the Faults Diagnosis

**Figure 16.**
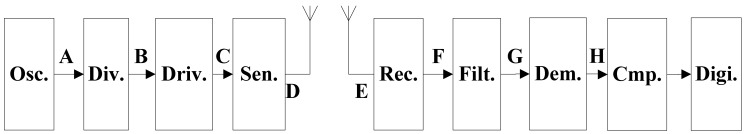
The block structure of the absolute positioning sensor.

The block structure of the absolute positioning sensor (one channel) can be expressed as seen in Figure 16.

The expected parameters and the error ranges that can be tolerated from A to H nodes are shown in Table 3.

The faults that are usually found in the A to C nodes are the amplitude reduction faults, constant output faults and frequency drift faults. The ones in D and E nodes are the resonance center drift faults and the low power faults. The ones in E to G are low power faults and constant output faults, and the fault in H node is a constant output fault.

For the signal amplitude reduction faults and the constant output faults, the synchronous clock is used to unify the specimens. Every specimen is a one-dimension vector with three times the period's length. For the frequency drifts and the resonance center drift faults, the FFT should be done to the sample points first, and then we select two times the center frequency length's one-dimension spectrum as a specimen. For all the faults above, the discriminant functions will be achieved by training the specimens. The data below is 10 one-dimension specimens collected from the FFT of the sending coils' waveforms, five are normal and the other five are in fault condition. Every specimen consists of 9 points which present the spectrums of the waveform. The data in detail is shown in Table 4.

After training the specimen above, the linear discriminant function is achieved. That is:

$$\begin{array}{l} y=\omega \cdot x+b_{0}\\ ={\left[-0.035,0.0415,0.1683,0.0998,-0.013,-0.0608,0.0013,-0.0492,-0.0416\right]}^{\prime }\cdot x-9.292 \end{array}$$

Finally, we substitute the test vectors into the discriminant function above, the exact rate is 97.3%.

## 5. Conclusions

In order to calibrate the accumulated error of the relative positioning sensor, the paper proposes an optimal design of the absolute positioning sensor for a high-speed maglev train. According to the difficulties we meet in the design and the debug for the sensor, the paper proposes a fault diagnosis method for the sensor. The signal flow method is used to locate the faults and the SVM theory is used to discriminate the faults for the sensor's analog circuit. Finally, the dependent relation of the former component and the latter component is used to reduce the error ratio after the fact. The results of the experiment show that the method is valid and reliable.

## Figures and Tables

**Table 1.** The results of the experiments on the test platform.

**Situation**	**Position mark plate binaries**	**The code the sensor reads out**				**Conclusion**

		**D_3_**	**D_2_**	**D_1_**	**D_0_**	
(**a**)	0001B	0	0	0	1	OK
	1111B	1	1	1	1	OK
	0101B	0	1	0	1	OK
	1001B	1	0	0	1	OK
(**b**)	0001B	0	0	0	1	OK
	1111B	1	1	1	1	OK
	0101B	0	1	0	1	OK
	1001B	1	0	0	1	OK
(**c**)	0001B	0	0	0	1	OK
	1111B	1	1	1	1	OK
	0101B	0	1	0	1	OK
	1001B	1	0	0	1	OK
(**d**)	0001B	0	0	0	1	OK
	1111B	1	1	1	1	OK
	0101B	0	1	0	1	OK
	1001B	1	0	0	1	OK

**Table 2.** The results of the experiments on experiment line.

**Group**	**Dir.**	**The value the sensor reads out/hex**											
1	Forward	110	111	112	113	114	115	116	117	118	119	11A	11B
	Reverse	11B	11A	119	118	117	116	115	114	113	112	111	110
2	Forward	110	111	112	113	114	115	116	117	118	119	11A	11B
	Reverse	11B	11A	119	118	117	116	115	114	113	112	111	110
3	Forward	110	111	112	113	114	115	116	117	118	119	11A	11B
	Reverse	11B	11A	119	118	117	116	115	114	113	112	111	110

**Table 3.** The expected characters.

**Nodes**	**Expected waveform**	**Amplitude/V**	**Frequency/kHz** *
A	square	5 ± 30%	2,000 ± 0.005%
B	square	5 ± 30%	2,000 ± 0.005%
C	square	5 ± 30%	2,000 ± 0.005%
D	sin	50 ± 20%	2,000 ± 0.005%
E	sin	0 to 5	2,000 ± 0.005%
F	sin	0 to 5	2,000 ± 0.05%
G	sin	4 ± 20%	2,000 ± 0.5%
H	DC	−4 to 4 DC	—

* Note: take one channel of the sensor for example.

**Table 4.** The specimen data of the FFT of the sending coils' waveforms.

	**1**	**2**	**3**	**4**	**5**	**6**	**7**	**8**	**9**
No fault 1	22	38	66	27.2	26.8	37.2	14.8	11.6	24
No fault 2	22	38.4	66	25.6	24.8	37.2	9.2	12	24.8
No fault 3	16.4	38.4	65.6	27.2	23.6	37.2	0	17.6	25.2
No fault 4	12	38	66	26.8	24.8	37.2	8.4	21.2	24.8
No fault 5	14.8	38	66	27.6	25.2	37.2	9.2	18.4	25.6
Fault 1	23.2	41.6	65.6	23.2	28	47.2	12	27.2	34.8
Fault 2	23.2	42	62.4	25.2	29.6	44.8	9.6	26	30.8
Fault 3	26.8	30.4	54.4	32	26.4	42	18.8	23.6	27.2
Fault 4	10.8	27.6	53.6	17.6	17.6	31.2	2.4	6.4	19.6
Fault 5	26.4	43.2	61.6	16.8	26.8	48.4	0	30	35.2
